# Proteins Associated with SF3a60 in *T. brucei*

**DOI:** 10.1371/journal.pone.0091956

**Published:** 2014-03-20

**Authors:** Benson Nyambega, Claudia Helbig, Daniel K. Masiga, Christine Clayton, Mariano J. Levin

**Affiliations:** 1 Laboratorio de Biología Molecular de la Enfermedad de Chagas, Instituto de Investigacíones en Ingeniería Genética y Biología Molecular (INGEBI), Buenos Aires, Argentina; 2 Molecular Biology and Biotechnology Department, International Center for Insect Physiology and Ecology (ICIPE), Nairobi, Kenya; 3 Zentrum für Molekulare Biologie der Universität Heidelberg (ZMBH), Heidelberg, Germany; Instituto Butantan, Laboratório Especial de Toxinologia Aplicada, Brazil

## Abstract

*Trypanosoma brucei* relies on Spliced leader *trans* splicing to generate functional messenger RNAs. *Trans* splicing joins the specialized SL exon from the SL RNA to pre-mRNAs and is mediated by the *trans*-spliceosome, which is made up of small nuclear ribonucleoprotein particles and non-snRNP factors. Although the trans spliceosome is essential for trypanosomatid gene expression, not all spliceosomal protein factors are known and of these, only a few are completely characterized. In this study, we have characterized the trypanosome Splicing Factor, SF3a60, the only currently annotated SF3a component. As expected, epitope-tagged SF3a60 localizes in the trypanosome nucleus. SF3a60 is essential for cell viability but its depletion seem to have no detectable effect on trans-splicing. In addition, we used SF3a60 as bait in a Yeast-2-hybrid system screen and identified its interacting protein factors. The interactions with SF3a120, SF3a66 and SAP130 were confirmed by tandem affinity purification and mass spectrometry.

## Introduction

*Trypanosoma brucei* is responsible for Human African Trypanosomiasis (HAT) and Animal African trypanosomiasis (AAT) in sub-Saharan Africa. This parasite relies on *trans* splicing where a common spliced leader (SL) is added to the 5′end of all immature mRNA transcripts to generate functional messenger RNAs. *Trans* splicing is mediated by the *trans*-spliceosome, which consists of small nuclear ribonucleoprotein particles (snRNPs) and non-snRNP factors (reviewed in [1], [2]). In higher eukaryotes, the assembly of the spliceosome involves both recognition of the 5′ splice site, the branch point and the 3′ splice site, mediated by U1 snRNP, branch-point-binding protein (SF1) and U2AF respectively. In mammals, the large subunit of U2AF recognizes the polypyrimidine tract while the small subunit binds to the 3′ splice site. Subsequently, the U2 snRNP is recruited and the U2 snRNA base pairs with the branch point sequence, displacing SF1 in a process mediated by the U2-associated protein complexes SF3a and SF3b. A similar sequence of events is thought to occur in trypanosomes, except that during *trans* splicing, the U1 snRNP is replaced by the spliced leader RNP (reviewed in [2]). In trypanosomes, the equivalents of all five mammalian small nuclear RNAs (snRNAs) and the corresponding snRNPs [3], [4] have been described; they deviate in several aspects from their human homologues. Systematic analyses of proteins harboring specific motifs [5]–[9], Tandem Affinity Purification (TAP-tagging) of splicing complexes coupled to mass spectrophotometric identification [2], [10] and *in silico* analyses [4] have enabled the identification and compilation of a list of spliceosomal proteins in *T. brucei*, including the trypanosome orthologues of all seven human SF3b components.

We focus here on trypanosome SF3a. In humans and yeast, SF3a contains three subunits: SF3a60 (yeast Prp9), SF3a66 (Prp11) and SF3a120 (Prp21), and each is essential for pre-splicing complex A formation [11], [12] and viability [12], [13]. Of these, only the SF3a60 subunit is currently annotated in the *T. brucei* genome database. We here functionally characterized *T. brucei* splicing factor SF3a60 and identified interacting protein partners via Yeast 2 Hybrid (Y2H) and TAP-tagging analyses.

## Methods

### Culture and Transfection

*Trypanosoma brucei* bloodstream and procyclic 427–1313 cells containing plasmid pHD 514 [18] were cultured in either HMI-9 medium for bloodstream cell lines [14] or MEM-PROS medium for procyclic cell lines [15], supplemented with 10% (vol/vol) fetal calf serum (Sigma-Aldrich). Cells were transfected as described previously [16].

### Cloning of TbSF3a60 and Identification of Proteins that Interact with *T. brucei* SF3a60 by Yeast Two-hybrid Screening

The coding sequence TbSF3a60 (TriTrypDB accession number Tb927.6.3160) was PCR amplified from 100 ng of total genomic DNA of *T. brucei* 927 strain using the primers SF3a forward, GGATCCAATGTTTGGTGGAGTTTTAGAAAAGAT bearing BamHI and reverse, TCAACCTGTTTTCCTCGACATCTG. The PCR product was cloned into pGEM-T (Promega), subsequently digested with *BamH*I and *Not*I and subcloned in frame into the ProQuest Yeast Two-Hybrid Gateway compatible System (Invitrogen). Bait cloning and Y2H screening were performed by Hybrigenics, S. A., Paris, France (http://www.hybrigenics.com/services.html). DNA from *Trypanosoma brucei* 927 strain, prepared by Dr. M. Turner, Glasgow Biomedical Research Centre, University of Glasgow, Scotland, UK, was randomly sheared and used to construct genomic library into the Y187 yeast strain. The library contained 7.5 million independent fragments, and was used for screening [17]. Twelve million interactions were actually tested with SF3a60. After selection on medium lacking Leu, Trp and His, 246 positive clones were picked from SF3a60 full-scale screen.

### Expression and TAP-tag Purification of *T. brucei* SF3a60

Procyclic trypanosomes expressing the tetracycline (*tet*) repressor from plasmid pHD1313 [18] were transfected with pHD 918 (tetracycline-inducible expression of the Tandem Affinity Purification (TAP) tag. The coding sequence of *SF3a60* (1629 bp) was amplified using primers CZ3371 (CGGGGCCCATGTTTGGTGGAGTTTTAGAAAAG (sense, *ApaI* site)) and CZ3372 (ATGTTAACACCTGTTTTC (antisense, *HpaI* site, no stop codon)) and genomic DNA of *T. brucei* strain Lister 427 as template. To express C-terminally TAP-tagged SF3a60, the open reading frame (ORF) was cloned in the *ApaI* and *HpaI* sites of pHD 918 [19]. Expression of the TAP tag alone and TAP-tagged TbSF3a60 was induced by adding 100 ng/ml tetracycline and cells were harvested 24 h later. Tandem affinity purified SF3a60 from procyclic trypanosomes [19] was separated on a 12% Sodium dodecyl sulphate (SDS-PAGE) and proteins stained with SYPRO Ruby following the manufacturer’s instructions (Invitrogen, Oregon, USA). The SF3a60 associated protein bands were excised and analyzed by nano-HPLC-electrospray ionization quadruple-time of flight-mass spectrometry (nano-HPLC ESI-QUAD TOF MS) and peaks identified as described elsewhere [20]. MS generated data were subjected to analysis with the Mascot software version 2.1.04.

### Western Blotting and Immunofluorescence of *T. brucei* Expressing the TAP-Tagged Proteins

Western blotting was performed as previously described [21]. Briefly, 2×10^6^ procyclic form *T. brucei* clones at log phase of growth were harvested, resuspended in Laemli buffer, subsequently separated on a denaturing 11% SDS-PAGE gel. Proteins were transferred to a nylon Hybond P membrane (Amersham Pharmacia), incubated with a 1∶50000 dilution of rabbit anti-Protein A antibody in transfer buffer and probed for expression of TbSF3a60-TAP using the peroxidase-anti-peroxidase complex (1∶50,000, Polysciences, Inc. - www.polysciences.com). Subsequently, clones expressing C-terminally TAP-tagged SF3a60 were harvested and the subcellular localization of the tagged SF3a60 determined by indirect immunofluorescence [22]. To detect the over-expressed SF3a60-TAP, 10^6^ trypanosomes were sedimented, re-suspended, then fixed in 4% paraformaldehyde (w/v) in 1x PBS for 18 min, sedimented again for 2 min, re-suspended in phosphate buffered saline (PBS), and allowed to settle down on a poly-lysine-coated glass surface for 25 min. Cells were blocked with 0.5% gelatin/PBS and thereafter incubated with a 1∶50000 dilution of rabbit anti-Protein A antibody (Sigma) for 1 h and with a 1∶500 dilution of the second antibody Alexa Fluor 594 goat a- rabbit IgG (Molecular 25 probes) for 40 min. The kinetoplast and the nuclear DNA were then stained with 100 ng/ml DAPI/1x PBS for 10 min.

### RNAi-mediated Silencing of *T. brucei* SF3a60

For RNAi in bloodstream forms, gene-specific fragments of ∼500 nt [23] were cloned into the vector p2T7TA blue [18] using the primers: 5′-GAGAAGATCTGCATGCGAGAAGCCACCTTCACTCGA–3′, 5′- CGGAATTCGTCGACGTTCCACCACATACCTCGCA–3′ for the 1629 bp SF3a60. In this vector, the RNA interference (RNAi) fragments are flanked by opposing T7 promoters with downstream tet operators. The resulting plasmids were linearized with Not I and transfected into bloodstream-form trypanosomes expressing the tet repressor (pHD 1313) and T7 RNA polymerase (pHD514) [18]. Transfectants were selected in 10 mg/ml hygromycin and cloned by limiting dilution. RNAi was induced by adding tetracycline to the medium at a concentration of 1 mg/ml. Samples were taken after every 24 hours of RNAi induction for three days.

### RNA Isolation and Northern Blotting

Total RNA was isolated from bloodstream *T. brucei* cell lines using Trifast (PeqLab Biotechnology GmbH). Total RNA (10 μg each) were separated on a denaturing formaldehyde agarose gel and blotted onto a Hybond N nylon membrane (Amersham Pharmacia). The *TbSF3a60, Tubulin (TUB) and SRP* DNA probes were labeled with [α32P]dCTP by random priming (Stratagene). After overnight hybridization, blots were subsequently washed in 1× SSC (0.15 M NaCl plus 0.015 M sodium citrate) and 0.1% sodium dodecyl sulfate (SDS) for 30 min at room temperature, in pre-warmed 1× SSC and 0.5% SDS for 45 min at 42°C, and in 0.1× SSC and 0.2% SDS for 30 min at 42°C before exposure to film phosphor imager.

### Primer Extension and Analysis of Splicing Intermediates

To assess the effect of *Tb*SF3a60 RNAi depletion on splicing, the amounts of *SLRNA* and Y-structure splicing intermediates were assessed by primer extension [24], [25]. RNA was prepared from the RNAi cell line grown without tetracycline, or with tetracycline for one or two days. Effectiveness of the RNAi was tested by growth inhibition when the cultures were maintained for a further day. Cells without tetracycline were also incubated with 2 μg/ml Sinefungin for 15 min. Total RNA was hybridized with either the spliced leader primer CZ2711 (5′-GCAGGAACCAACAGCACAATGCG-3′) or a U3 snRNA (primer CZ2712 5′-TGCCGTTCATCGAAC-3′) before incubation with reverse transcriptase and dNTPs, including radioactive dCTP. The products were run on a 6% polyacrylamide/urea gel with 32P-labelled DNA markers and detected by phosphorimaging.

## Results

### SF3a60 is in the Trypanosome Nucleus

**Figure 1:**
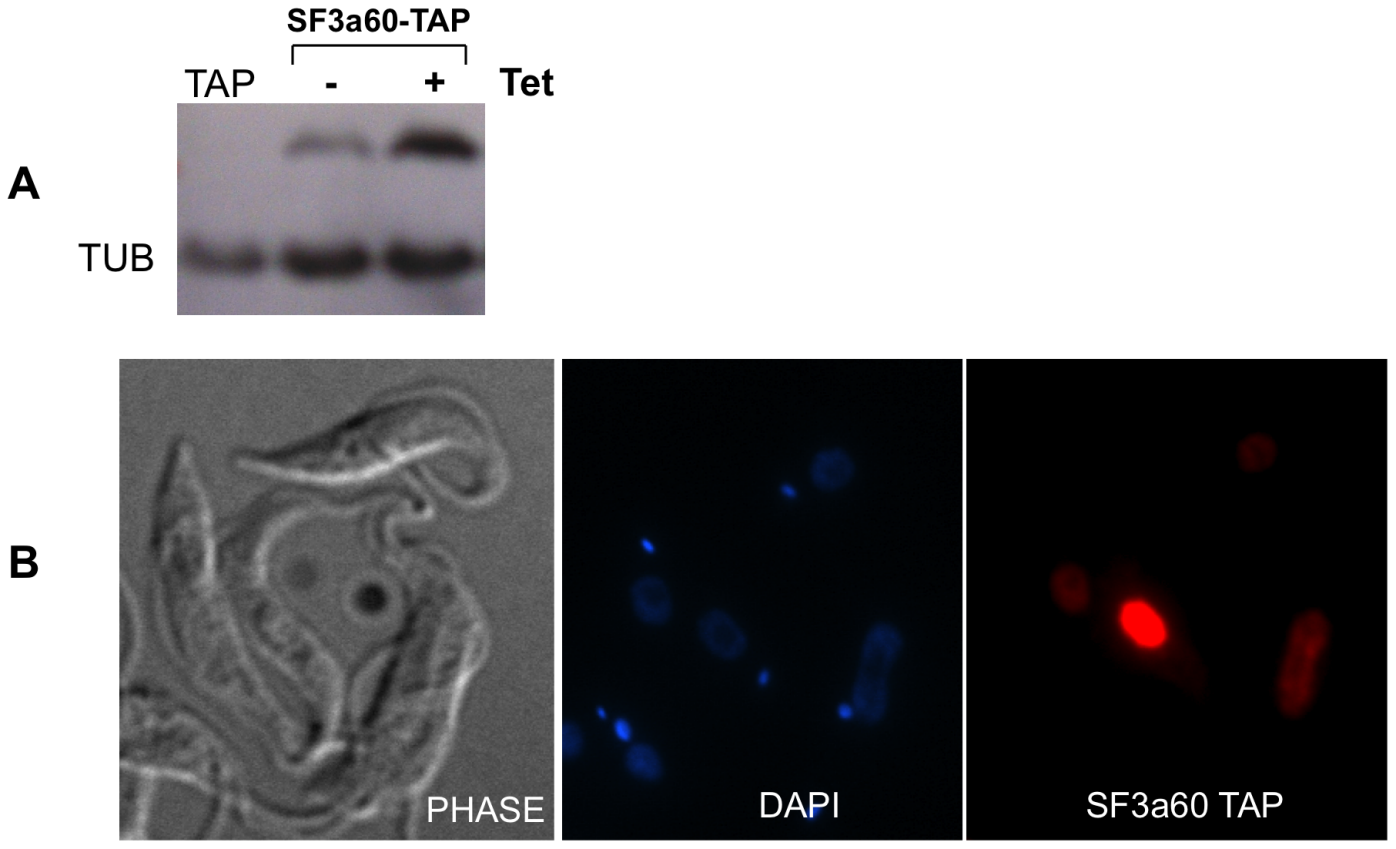
Trypanosome SF3a60 localizes in the nucleus. (A) Cells expressing *SF3a60*-TAP and the TAP tag with (+) and without (−) tetracycline induction after Western blot analyses. The SF3a60-TAP is conditionally expressed while tubulin (TUB) expression is used as control and (B). Immunofluorescence of procyclic form trypanosomes TAP-tagged *TbSF3a60* counterstained for *Tb*SF3a60 (red) and for DNA (DAPI).

Trypanosome SF3a60 (Tb927.6.3160) is already annotated as such in the genome database, although without experimental evidence. To determine its location, we expressed SF3a60 with a C-terminal TAP-tag in procyclic trypanosomes (Figure 1A). Cells expressing the tagged protein grew normally and SF3a60-TAP was detected exclusively within the nucleus (Figure 1B) although unequivocal evidence would require the detection of endogenous SF3a60 via immunofluorescence with anti-SF3a60 antibody.

### SF3a60 Silencing Affects Trypanosome Viability with no Detectable Trans Splicing Defects

**Figure 2:**
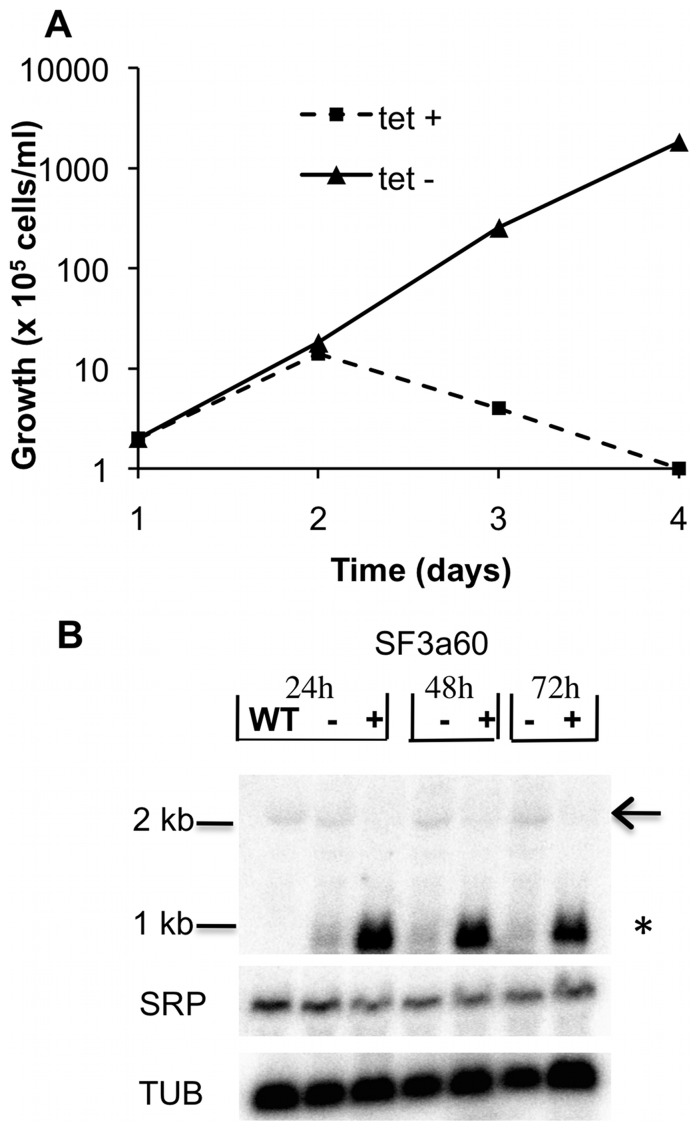
S3a60 is essential for trypanosome viability. (A) Cumulative growth curves of TbSF3a60 depleted trypanosomes. The cells grown with (triangles, solid line) or without (squares, dashed line) tetracycline. Every 24 h, samples were taken for the determination of cell density (cells/ml), and grown cultures were diluted down to 5×10^5^ cells/ml with fresh medium. (B). Effect on *SF3a60* mRNA. Cells were grown either without *Tet* (–), or in the presence of 100 ng/ml *Tet* (+) for 24, 48 and 72 hours. Each lane on the Northern blot contains 10 μg RNA from bloodstream form wild type (WT) or an RNAi cell line. Asterisk (*) denotes dsRNA. Trypanosome SRP and Tubulin (TUB) RNAs are used as loading controls. Arrow indicates SF3a60 mRNA.

**Figure 3:**
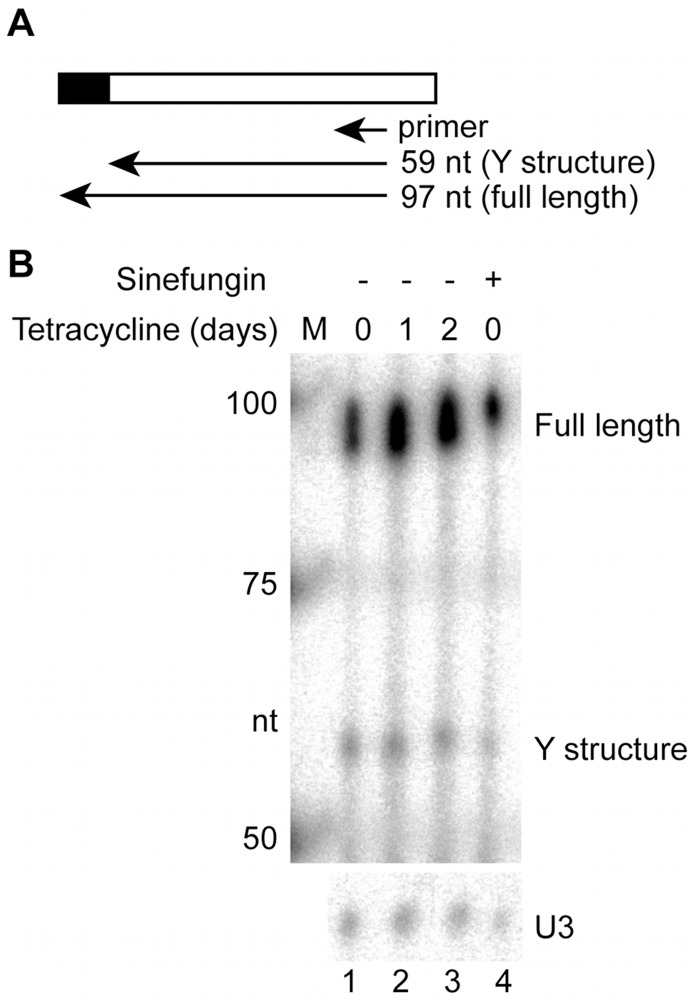
SF3a60 RNAi has no effect on splicing. The Y structure *trans* splicing intermediate is not decreased after two days of RNAi targeting SF3a60. The full-length spliced leader RNA and the Y structure were detected by primer extension (diagram in panel A) followed by denaturing polyacrylamide gel electrophoresis (B) with the U3 snRNA as a loading control. The RNA was prepared from the RNAi cell line grown without tetracycline (panel B, lanes 1 and 4), or with tetracycline for one or two days (lanes 2 and 3). M: markers.

The down regulation of SF3a60 expression using RNA interference resulted in growth inhibition within 48 hours (Figure 2A) and efficient reduction in the SF3a60 mRNA (Figure 2B). To test whether the RNAi gave detectable inhibition of splicing, we chose a time point when there was not yet severe growth inhibition. Northern blotting using a tubulin probe revealed the presence of a bicistronic beta-alpha tubulin RNA (Figure 2B), the abundance of which is increased on splicing inhibition [25]. We observed no increase in this bicistronic RNA after SF3a60 RNAi. In addition, we used primer extension to measure the abundance of the Y-structure trans splicing intermediate. As expected, incubation with Sinefungin caused a decrease in the amount of Y structure relative to full-length *SLRNA*, and the migration of the full-length primer extension product was very slightly slower than normal, due to a decrease in cap methylation [25] (Figure 3: lane 4 against lane 1). In contrast, RNAi (lanes 2 and 3) reproducibly had no effect on the migration of the full-length *SLRNA* or the relative abundance of the Y structure. It is evident that although SF3a60 is essential, RNAi does not cause a detectable decrease in splicing, at least under conditions when growth inhibition is not yet detected.

### Components of the Trypanosome SF3a Complex are Conserved

**Figure 4:**
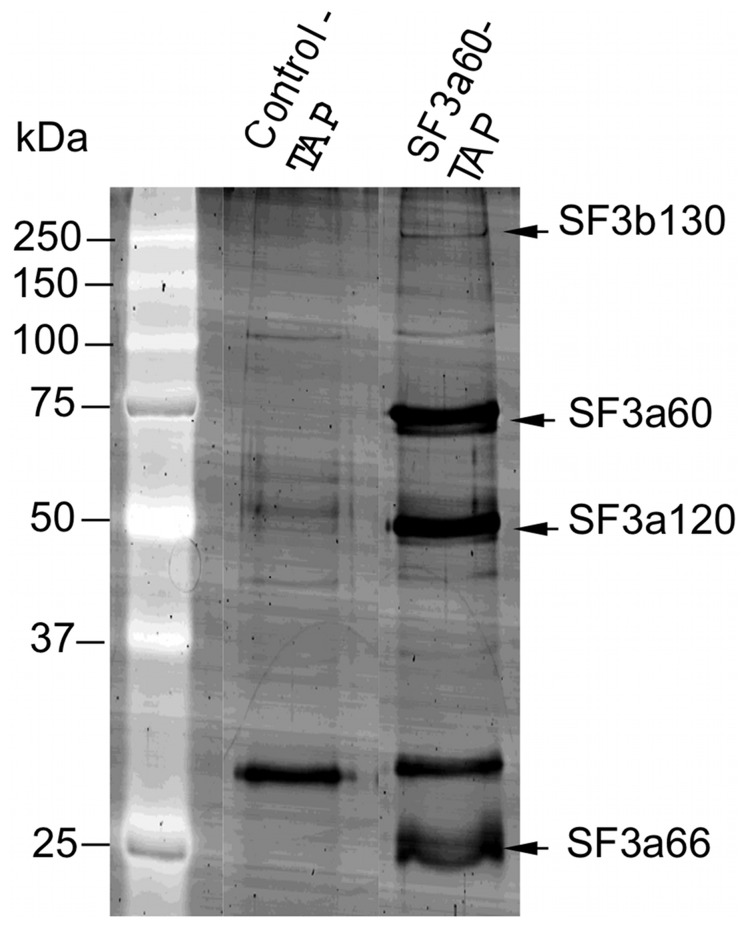
Preparative tandem affinity purification identifies SF3a60 associated proteins. Proteins were separated on 12% SDS-PAGE and stained with SYPRO Ruby. Marker protein sizes in kilo Daltons are indicated on the left and corresponding protein bands are on the right. Details are in Table 1.

**Figure 5:**
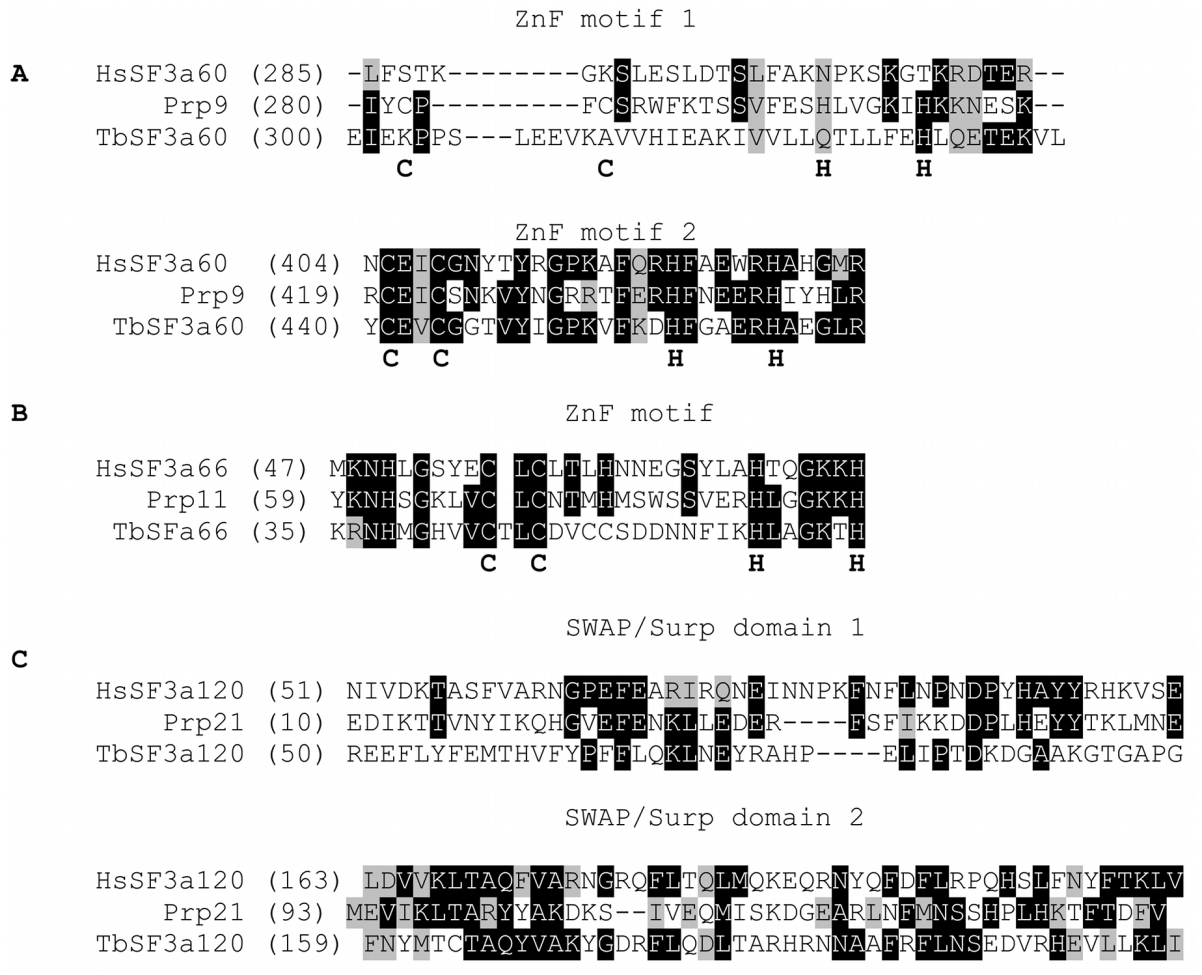
C2H2 and SWAP/Surp domains are also conserved in *T. brucei* SF3a subunits. Black and light grey shading indicate identical and conserved amino acid residues, respectively. **A**. *T. brucei*, *Homo sapiens* (AAH11523.1) and *Saccharomyces cerevisiae* (CAA98589.1). Human, yeast and *T. brucei* have each, a C-terminal C2H2-like Zn finger motif while yeast has a second N-terminal C2H2-like motif. **B**. *T. brucei*, *Homo sapiens* (AAH09903.1) and *Saccharomyces cerevisiae* (CAA98602.1). Both human, yeast and trypanosome have a common Cys2His2-like Zn finger motif. **C**. *T. brucei*, *Homo sapiens* (AAH01976.1) and *Saccharomyces cerevisiae* (CAA89497.1). The human and yeast sequences have two SWAP/Surp domains each in tandem while *T. brucei* has just one SWAP/Surp domain.

To identify the components of SF3a we purified it using C-terminally TAP-tagged SF3a60. Purification of an empty TAP-tagged construct served as a control. Figure 4 shows the SYPRO Ruby stained protein gel and the indentified proteins are listed in Table 1. We found four proteins with 5 or more significant peptide matches. Tb927.3.1140 and Tb927.2.2270, the homologues of SF3a120 and SF3a66, were clearly identified. It is therefore possible that the trypanosome SF3a60 can interact with these components individually and it will require further experiments to demonstrate they form a single complex in trypanosomes. Figure 5 shows the partial sequence alignments of trypanosome, human and yeast SF3a subunits. *T. brucei* SF3a60, like protein homologues from human [26] and *S. cerevisiae* [27], share considerable homology in their C-terminal zinc finger domain. However, only the *S. cerevisiae* SF3a60 contains a second C_2_H_2_ zinc finger domain in its central region. This second zinc finger has also been noted in *S. pombe*, *N. crassa* [27] (data not shown). SF3a66 proteins from *T. brucei* (accession no. XP951536), human [28], and *S. cerevisiae* [29] all harbor a conserved C_2_H_2_ zinc finger domain. The C-terminal sequence of *T. brucei* and yeast are relatively shorter and lack the proline-rich heptapeptide repeats [28]. Trypanosome SF3a120 (accession no. XP843708), unlike human [30] and *S. cerevisiae* [31] which have SURP-1 and SURP-2 domains in tandem, contain only one C-terminal SURP-2 domain and an evidently shorter C-terminal segment. As expected, the SURP-2 domain is within the selected interaction domain (Figure S1).

**Table 1:** Proteins associated with SF3a60 by tandem affinity purification.

MS identified *T. brucei* protein	MW (Da)	Score	PEP	% Coverage	Homologues
Tb927.7.6980 **(SF3b130)**	198106	99	5	1.5	Rse1 (yeast), SF3b130 (human)
Tb927.6.3160 **(SF3a60)**	62262	1024	142	39	Prp9 (yeast), SF3a60 (human)
Tb927.3.1140 **(SF3a120)**	43679	2952	122	39	Prp21 (yeast), SF3a120 (human)
Tb927.2.2270 **(SF3a66)**	26890	72	25	9.6	Prp11 (yeast), SF3a66 (human)

In addition, we identified Tb927.7.6980. Although this protein is not yet annotated, it is clearly SF3b130 as judged both by BLAST searches (score and coverage shown in Table 1) and the fact that it was previously shown to co-purify with tagged SmD1 [10]. The SF3b subunits, in contrast, were not found in those experiments.

### Genome-wide Yeast Two-hybrid (Y2H) Screening Identifies Novel Potential SF3a60 Interaction Partners

**Figure 6:**
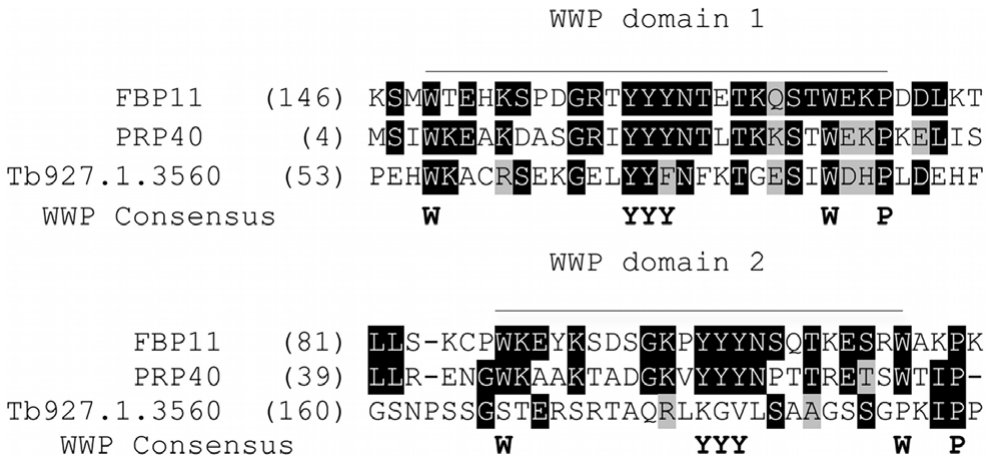
Sequence conservation for putative T. brucei PRP40. **A**. The WW domains of human FBP11 (O75400.2), yeast PRP40 (NP_012913.3) and the putative Trypanosome FBP11 homologue contain the tryptophans, prolines and a central set of three hydrophobic residues characteristic of WW domains. Evidently, the putative *T. brucei* FBP11 lacks a second WW domain.

Tandem affinity purification normally results only in the detection of stable interactions. To see if we could trace any other interactions, which might be sub-stoichiometric or transient, we screened a Yeast 2 hybrid *T. brucei* prey genomic library using SF3a60 as bait. Given the enormous evolutionary distance between yeast and trypanosomes, indirect interactions bridged by specific binding to yeast spliceosome components were not expected. The screen led to the identification of 44 possible protein preys (excluding self-activating and redundant hits). Using a predicted biological score (PBS), calculated according to [32], the protein preys were grouped into four categories: very high confidence, high confidence, good confidence and moderate confidence (Summarized in Table 2). The domains and portion of each protein hit that mediates the interaction with SF3a60, the so-called selected interaction domain (SID) has also been shown (Figures S1 to S4). Reassuringly, SF3a120 was clearly identified as a very high confidence prey; among the others in this category were a putative splicing-associated RNA helicase and two TPR repeat proteins. The 24 “moderate” confidence interactions included the two genes encoding the RNA polymerase II largest subunit RPB1 [33]. Tb927.10.15570, a putative transcription factor [34] was also identified in this screen. Local sequence alignment using EMBOSS (www.ebi.ac.uk/Tools/psa/) revealed that Tb927.1.3560 is 18% identical and 29.9% similar to the yeast U1 snRNP component Prp40; it contains WW domains, two highly conserved tryptophans that bind proline-rich peptide motifs found in the splicing factor Prp40 [35], [36] but no FF domains (Figure 6, Figure S5). SF3b components were not found, suggesting that the interaction between SF3a60 and SF3b is indirect or via the core U2 snRNP [11].

**Table 2:** SF3a60-associated protein preys from a genome-wide Yeast-2-hybrid screen.

1Confidence Level	TriTrypDB locus No.	2Domain/Motif	3Annotation	Source/Ref
Very high	Tb927.11.1520	Coiled domain	Expression site-associated gene (ESAG3) protein, putative.	TritrypDB
	Tb927.11.16890	TPR	Hypothetical protein, conserved	TritrypDB
	Tb927.11.4550	Coiled domain	Hypothetical protein, conserved	TritrypDB
	Tb927.2.2210		Hypothetical protein, conserved, mitochondrial	TritrypDB
	Tb927.3.1140	SWAP/Surp	*T. brucei* SF3a120	This study
	Tb927.3.3150	TPR	Hypothetical protein, conserved	TritrypDB
	Tb927.7.1560	TPR	(Yeast TOM71 (P = 2.6e-07), blast-back P = 3.6e-07)	[68]
	Tb927.7.4410		(Yeast CAF120 (P = 0.68), blast-back P = 0.86)	[69]
	Tb927.10.7280	DEAD/DEAH	(Yeast PRP22 (P = 2.9e-173), blast-back P = 2.1e-178)	[50] [51]
High	Tb927.9.11930	Rad4	*T. brucei* DNA repair protein, putative)	TritrypDB
	Tb927.10.8130	TM	Hypothetical protein, conserved	TritrypDB
	Tb927.11.2290		Hypothetical protein, conserved	TritrypDB
	Tb927.1.3560	WW (but no FF)	(Yeast PRP40 (P = 0.015), blast-back P = 0.0026)	[35] [36]
	Tb927.3.4460		(Yeast SRB2 (P = 0.77), blast-back P = 0.88)	[70]
Good	Tb927.7.4080	ATPase, helicase	*T. brucei* DNA excision repair protein	TritrypDB
	Tb11.01.6840	Coiled domain	Hypothetical protein, conserved	TritrypDB
	Tb927.11.16580	TPR	Hypothetical protein, conserved	TritrypDB
	Tb927.11.16990	Nucleotide hydrolase	Hypothetical protein, conserved	TritrypDB
		GTPase		
	Tb927.11.3410	TM	Hypothetical protein, conserved	TritrypDB
	Tb927.3.3130	TM, SAM	Hypothetical protein, conserved	TritrypDB
Moderate	Tb927.4.5020	Rpb1, C2H2	*T. brucei* RNA polymerase IIA largest subunit	TritrypDB
	Tb927.8.7400	Rpb1, C2H2	*T. brucei* RNA polymerase IIA largest subunit	TritrypDB
	Tb927.8.930	Coiled domain	Hypothetical protein, conserved	TritrypDB
	Tb927.9.3680	Prefoldin superfamily,	Hypothetical protein, conserved	TritrypDB
		Coiled domain		
	Tb927.9.10840		Hypothetical protein, conserved	TritrypDB
	Tb927.10.12870	Coiled domain	Hypothetical protein, conserved	TritrypDB
	Tb927.10.15570	Coiled domain	*T. brucei* transcription factor IIA-2	[34]
	Tb927.10.5910	Coiled domain	Hypothetical protein, conserved	TritrypDB
	Tb927.10.5400	TM, coiled domain	Hypothetical protein, conserved	TritrypDB
	Tb927.10.2600		Hypothetical protein, conserved	TritrypDB
	Tb927.11.16050	TPR, HSP20	(Hsp90 cochaperone, (P = 0.00023), blast-back P = 0.00059)	[71]
	Tb927.11.5410		Hypothetical protein, conserved	TritrypDB
	Tb927.3.3220	TPR	*T. brucei* CTR9	[56]
	Tb927.3.3810	2xTM	Hypothetical protein, conserved	TritrypDB
	Tb927.4.2640	TPR, IQ Calmodulin	Hypothetical protein, conserved	TritrypDB
	Tb927.5.4110	ARM repeat	(Yeast RTG2 (P = 0.37), blast-back P = 0.49)	[72]
	Tb927.6.900	Coiled, ApoLp-III-like	Hypothetical protein, conserved	TritrypDB
	Tb927.7.2330	DUF1663	Hypothetical protein, conserved	TritrypDB
	Tb927.7.4150		Hypothetical protein, conserved	TritrypDB
	Tb927.7.5430		Hypothetical protein, conserved	TritrypDB
	Tb927.8.2880		Hypothetical protein, conserved, mitochondrial.	TritrypDB
	Tb927.8.4400		Hypothetical protein, conserved	TritrypDB
	Tb927.8.4870	WD40	*T. brucei* CMF6, DIGIT, flagellar component	[73]
	Tb927.8.8200	PIN domain, prefoldin	Hypothetical protein, conserved	TritrypDB

1 Confidence levels according to PBS scores [32].

2 Domains (superfamily, Pfam) predicted in TritrypDB (http://tritrypdb.org/tritrypdb/); a blank space indicates those without known domains.

3 Functional designation. Functions predicted on the basis of sequence match alone are shown in parentheses.

## Discussion

We have here presented experimental evidence identifying potential trypanosome homologues to yeast Prp9 (human SF3a60), Prp21 (human SF3a120) and Prp11 (human SF3a66). Compared to the human and yeast homologues, the trypanosome SF3a proteins show N-terminal sequence divergence but as shown in figure 5, contain all the functional domains which in humans, are needed to facilitate interactions between themselves and for integration into the U2 snRNP [11]. Concurrent with data from previous studies in which SF3a subunits from human and yeast readily formed the SF3a heterotrimer [11], [28], [30], [37]–[40], our TAP-tagging results demonstrate the association between SF3 a60, SF3 a66 and SF3 a120 in the trypanosome.

Although the Northern blots of SF3a60-depleted cells showed reduced amounts of mature SF3a60 mRNA as expected, we detected no effect on trans-splicing (Figure 3). In addition, we saw no accumulation of tubulin mRNA dimers suggesting that tubulin processing and stability [41]–[43] was not compromised, which could also imply trypanosome SF3a60 RNAi has no effect on pre-mRNA splicing. There are several possible explanations for this. The first is that this is in fact not SF3a60: this is unlikely based on co-purification with other proteins similar to SF3a components. The second is that SF3a60 is not required for trans splicing, but as a scaffold for other splicing factors which is improbable, but not impossible. Already, there are instances in other eukaryotes where splicing factors have been found essential for spliceosome assembly and consequently cell viability, but not for splicing [44], [45]. The third explanation is that upon sufficient depletion, the inhibition of RNA processing is so severe that it rapidly results in cell death.

In the TAP purification, the excised SF3a60 band was actually a doublet (Figure 4), with a slightly faster-migrating component that appeared to be present at sub-stoichiometric levels; we do not know what this is - it could be modified SF3a60, but could also be HSP70, which was (as always) identified as a contaminant. A much slower-migrating band was identified as (SAP130) SF3b130. Other components of SF3b may also have been present in our purification at sub-stoichiomentric levels, but were not identified because we sequenced only strongly stained bands. An exhaustive two-hybrid screen previously identified the physical interaction between *S. cerevisiae* Prp9p and Rse 1p, the yeast homologue of SF3b130 [46]. Recently, a high throughput affinity-capture experiments with Rse1p detected interaction with all three SF3a components [47], [48].

To detect sub-stoichiometric or less stable interactions, we conducted a yeast two-hybrid screen using SF3a60 as bait. The differences with respect to number of hits identified between TAP pull-down and two hybrid screens is striking, but expected. Although likely to be transient, these hits were nonetheless biologically significant and are likely to occur in vivo and could be validated further via experimental functional analyses. While we identified hits across all the Global PBS confidence intervals, several of these proteins have domains that suggest involvement in aspects of gene regulation. An interaction between human SF3a120 and SF3a60 was previously documented [11] and, reassuringly, was confirmed here for trypanosomes, documenting the sensitivity of our screen. This interaction was detectable despite the presence of only one C-terminal SURP-2 domain in trypanosome SF3a120. The SURP2 domain has previously been implicated in protein binding activity to SF3a60 [49]. SF3b130 was not, in contrast, found in the yeast two-hybrid screen. Although this result may be a false negative, it is likely SF3b associates with SF3a via a subunit other than SF3a60. The specificity of the remaining interactions will require verification. We identified two putative helicases: Tb927.10.7280 (a DExD/H box protein that is already annotated as a putative splicing factor, although without experimental evidence) and Tb927.7.4080. Their corresponding homologues in yeast (Prp22 and RAD26) are involved in pre-mRNA splicing [50], [51] and DNA repair [52] respectively. The assignment of Tb927.10.7280 as a pre-mRNA splicing factor in the Tritryp database is however without any experimental evidence and its association with *T. brucei* SF360 is a positive step towards its complete annotation. Six SF3a60 associated proteins have TPR motifs, which predisposes them to various aspects of protein-protein interactions, including during splicing [53]–[55] and are candidates for further characterization. One of these, Tb927.3.3220 (trypanosome CTR9) has recently been shown to interact with trypanosome CDC73 and a putative LEO1 homolog [56]. In Opisthokonts and plants, these proteins are components of the Paf complex, which functions in different aspects of gene expression including transcriptional elongation and termination, mRNA processing and export and epigenetic changes [57]. The mitochondrial membrane localization for Tb927.2.2210 and Tb927.8.2880 is interesting and could be subject to further investigation, especially since a group of yeast mitochondrial proteins have been associated with T6, one of the six complexes primarily populated by spliceosomal proteins [58].

One of the high-confidence interaction partners, Tb927.1.3560, could be the putative trypanosome homologue of yeast Prp40, a component of the U1 snRNP - although it was not found to associate with tagged TbU1–70K [59] or the core snRNP protein SmD1 [10], SmB [60] and SmD3 [61] in proteomic analyses. The U1 snRNP is not engaged in *trans* splicing, but is required for the *cis* splicing of at least two trypanosome mRNAs [62]. Although the putative trypanosome FBP11 homologue has only one of the two N-terminal WW domains found in corresponding yeast and human sequences (Figure 6) and lacks the associated FF repeats (Figure S5), previous studies have shown that Prp40 WW domains are sufficient for binding to the phospho-CTD (C-terminal repeat domain) and that the FF regions could function to increase the overall phospho-CTD binding affinity [35]. A possible role of Prp40 in U2 snRNP – E complex association [36] has been suggested previously. That Tb927.1.3560 is the trypanosome homologue to yeast Prp40 could extend our understanding of a possible association of U1 snRNP and the recruitment of U2 snRNP in trans-splicing. U2 snRNP specific proteins (A′, B′ and SF3a/b sub-units) were also found at low levels in association with the E complex [63]. Tb927.10.15570 was also identified in this study as potential prey to SF3a60. This protein was previously identified as an as an integral component of the RNA polymerase II transcription factor complex that contains SNAPc, TRF4/TBP and TFIIA-1 and consequently named TbTFIIA-2 [34]. TRF4/TBP-SNAPc-TFIIA binds to the SL RNA gene promoter and is absolutely essential for SL RNA gene transcription [34], [64].

The most interesting 2-hybrid interaction was with the largest subunit of RNA polymerase II (RPB1). There is a voluminous literature describing the strong link between polymerase II transcription and splicing in metazoans and yeast (reviewed in [65]). The observation of a direct interaction between SF3a and RNA polymerase II is surprising for two reasons. First, most papers concerning eukaryotic *cis* splicing discuss mainly an association between the spliceosome and the phosphorylated C-terminal heptad repeats of RNA polymerase II (reviewed in [66]). The trypanosome RNA polymerase II lacks heptad repeats and it is unknown whether it can be phosphorylated in yeast. Second, the association between polymerase II transcription and splicing appears to occur mainly during events at the 5′ splice site (summarized in [67]). Evidence so far indicates that in trypanosomes, events at the *trans* splicing acceptor site - in which SF3 is involved - are independent of the transcribing polymerase [67]. Nevertheless, the possible interaction between trypanosome RNA polymerase II and SF3a, in addition to the cellular roles of the detected hypothetical gene products deserve further investigation.

## Supporting Information

Figure S1 Domain organization of very high confidence SF3a60 associated proteins. The hits, their corresponding domains and the amino acid portion involved in the interaction with SF3a60 (SID) are shown.
(PDF)

Figure S2 Domain organization of high confidence SF3a60 associated proteins. The hits, their corresponding domains and the amino acid portion involved in the interaction with SF3a60 (SID) are shown.
(PDF)

Figure S3 Domain organization of good confidence SF3a60 associated proteins. The hits, their corresponding domains and the amino acid portion involved in the interaction with SF3a60 (SID) are shown.
(PDF)

Figure S4 Domain organization of moderate confidence SF3a60 associated proteins. The hits, their corresponding domains and the amino acid portion involved in the interaction with SF3a60 (SID) are shown.
(PDF)

Figure S5 The FF domains of PRP40 proteins. The corresponding FF domains of human FBP11 (O75400.2), yeast PRP40 (NP_012913.3) are shown here in bold and starting points marked with an asterisk (*) within the alignment, except for *T. brucei* FBP11 homologue where they are absent.
(PDF)
