# Supplementary figures and images for: Proteins Associated with SF3a60 in T. brucei
Source: PLoS One. 2014 Mar 20;9(3):e91956. doi: 10.1371/journal.pone.0091956 (PMC3961280; doi:10.1371/journal.pone.0091956)

Figure S1

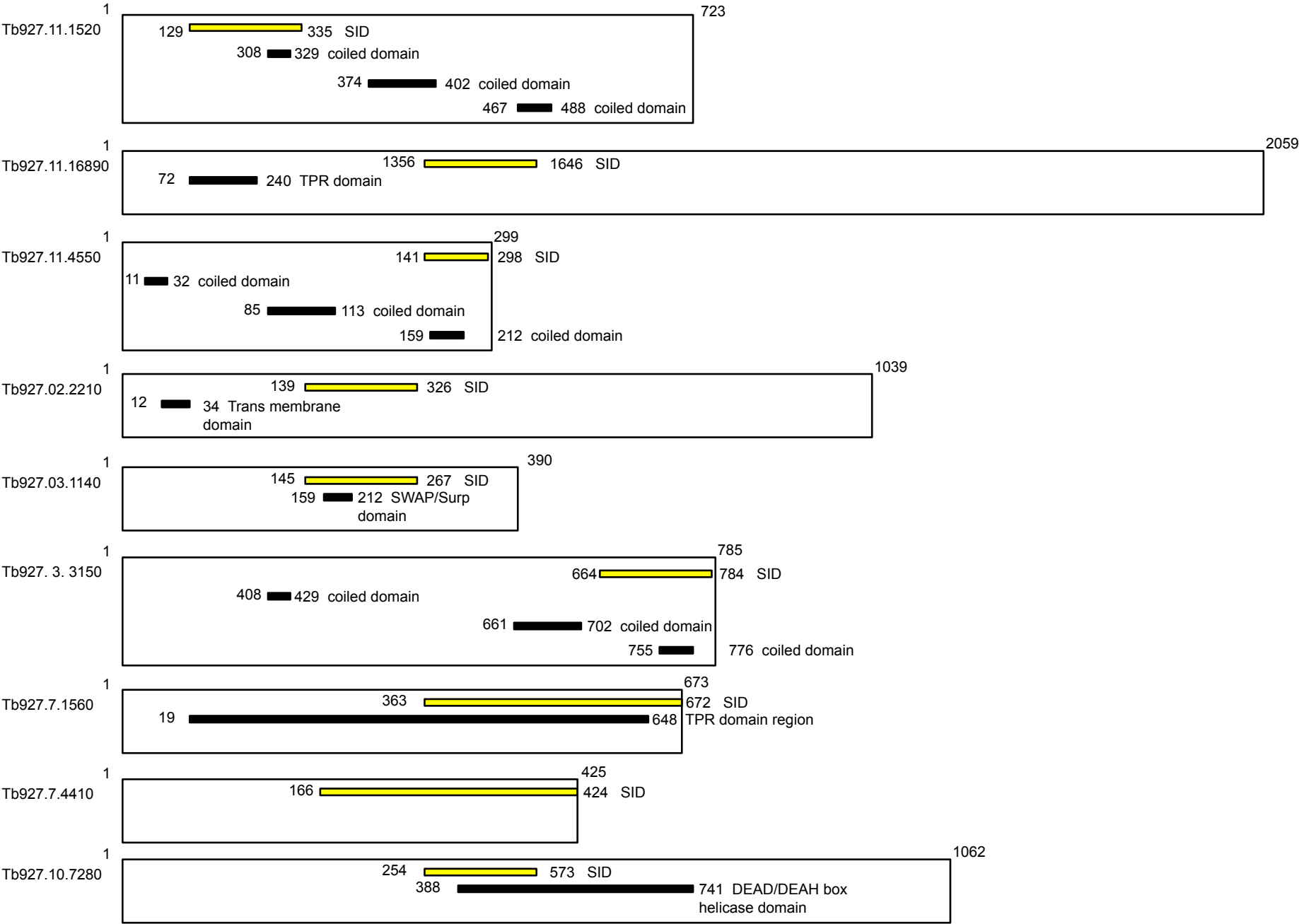

Supplement: Figure S1 — Domain organization of very high confidence SF3a60 associated proteins. The hits, their corresponding domains and the amino acid portion involved in the interaction with SF3a60 (SID) are shown. (PDF) [file pone.0091956.s001.pdf]

Figure S2

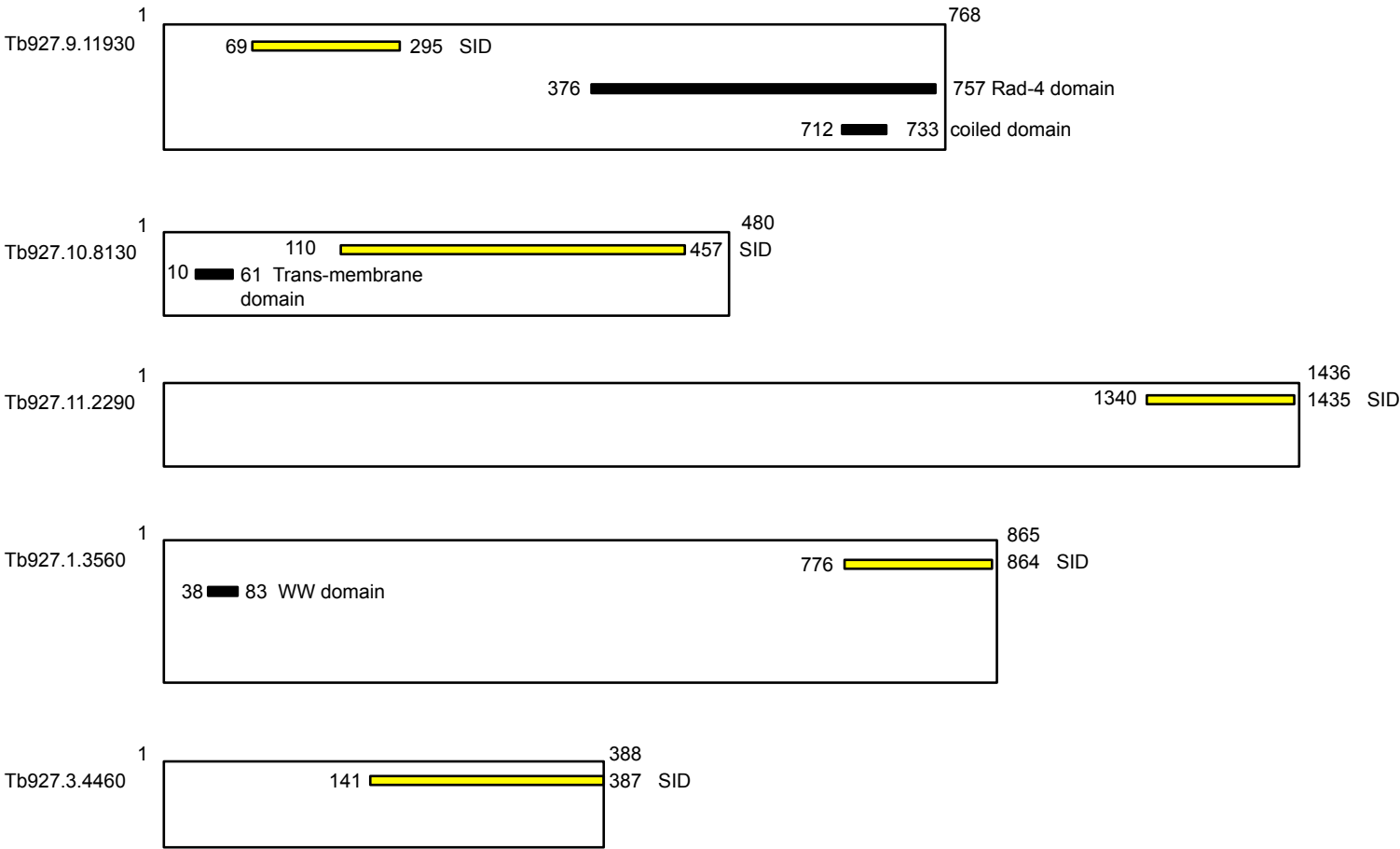

Supplement: Figure S2 — Domain organization of high confidence SF3a60 associated proteins. The hits, their corresponding domains and the amino acid portion involved in the interaction with SF3a60 (SID) are shown. (PDF) [file pone.0091956.s002.pdf]

Figure S3

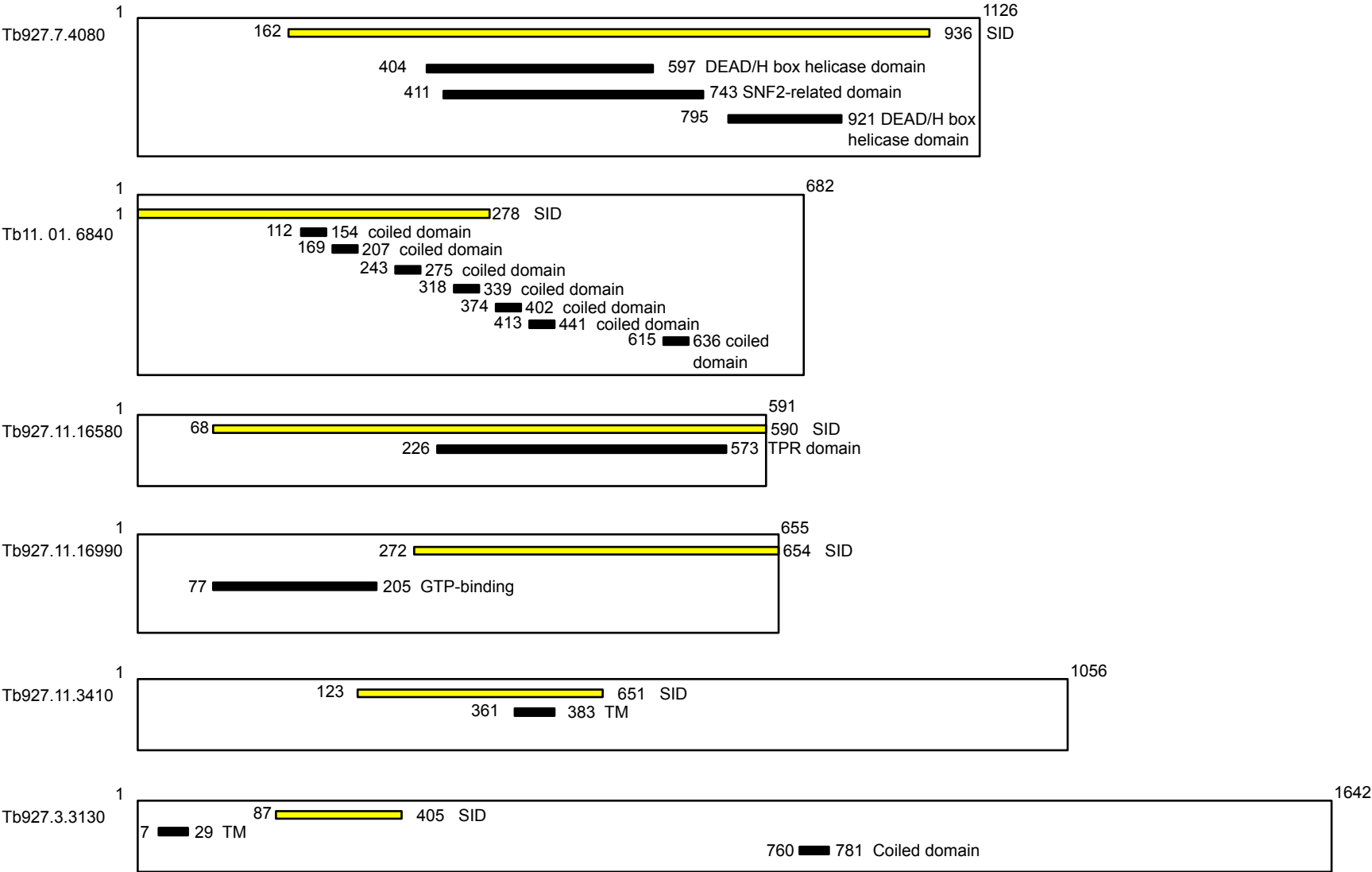

Supplement: Figure S3 — Domain organization of good confidence SF3a60 associated proteins. The hits, their corresponding domains and the amino acid portion involved in the interaction with SF3a60 (SID) are shown. (PDF) [file pone.0091956.s003.pdf]

Figure S4

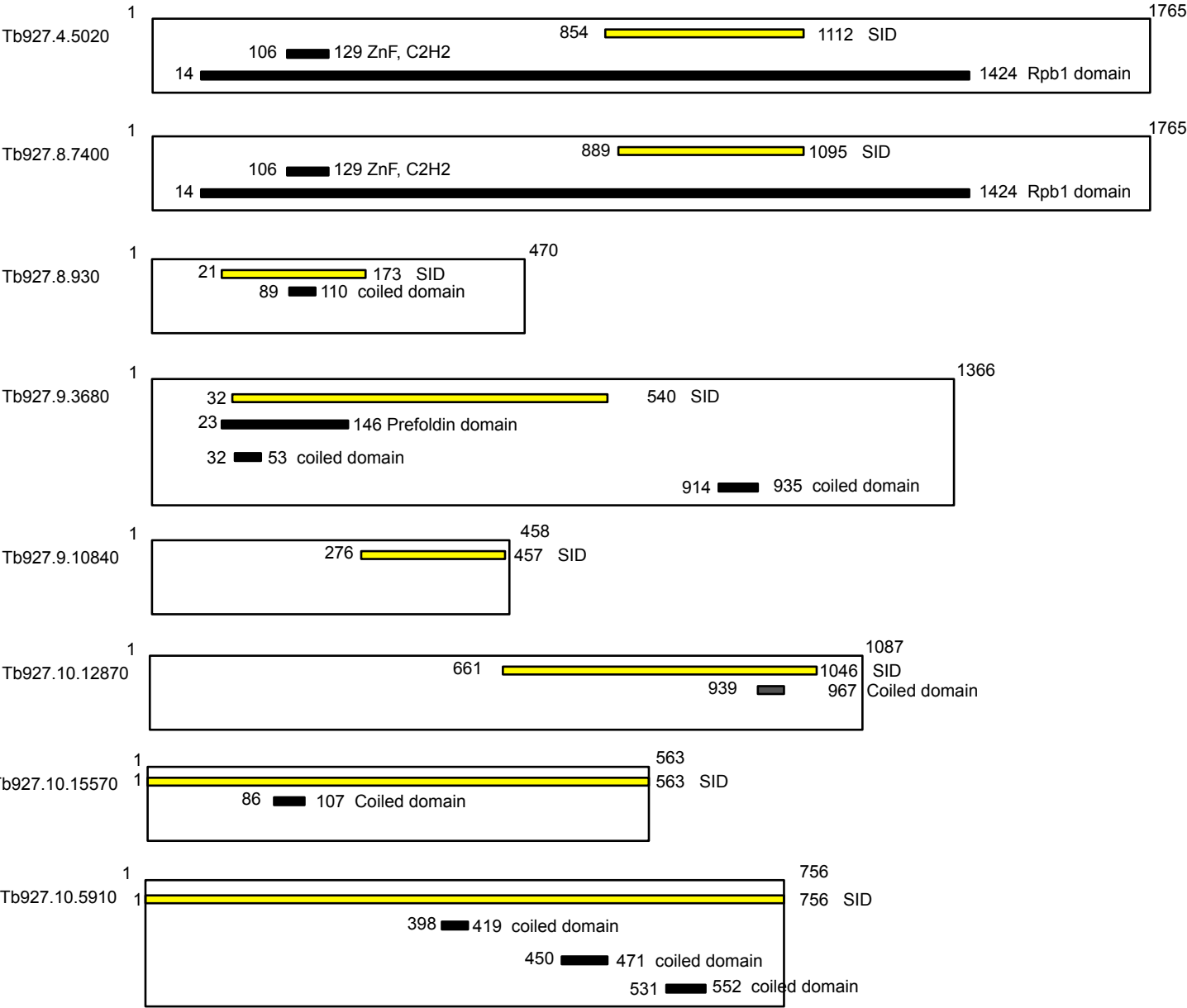

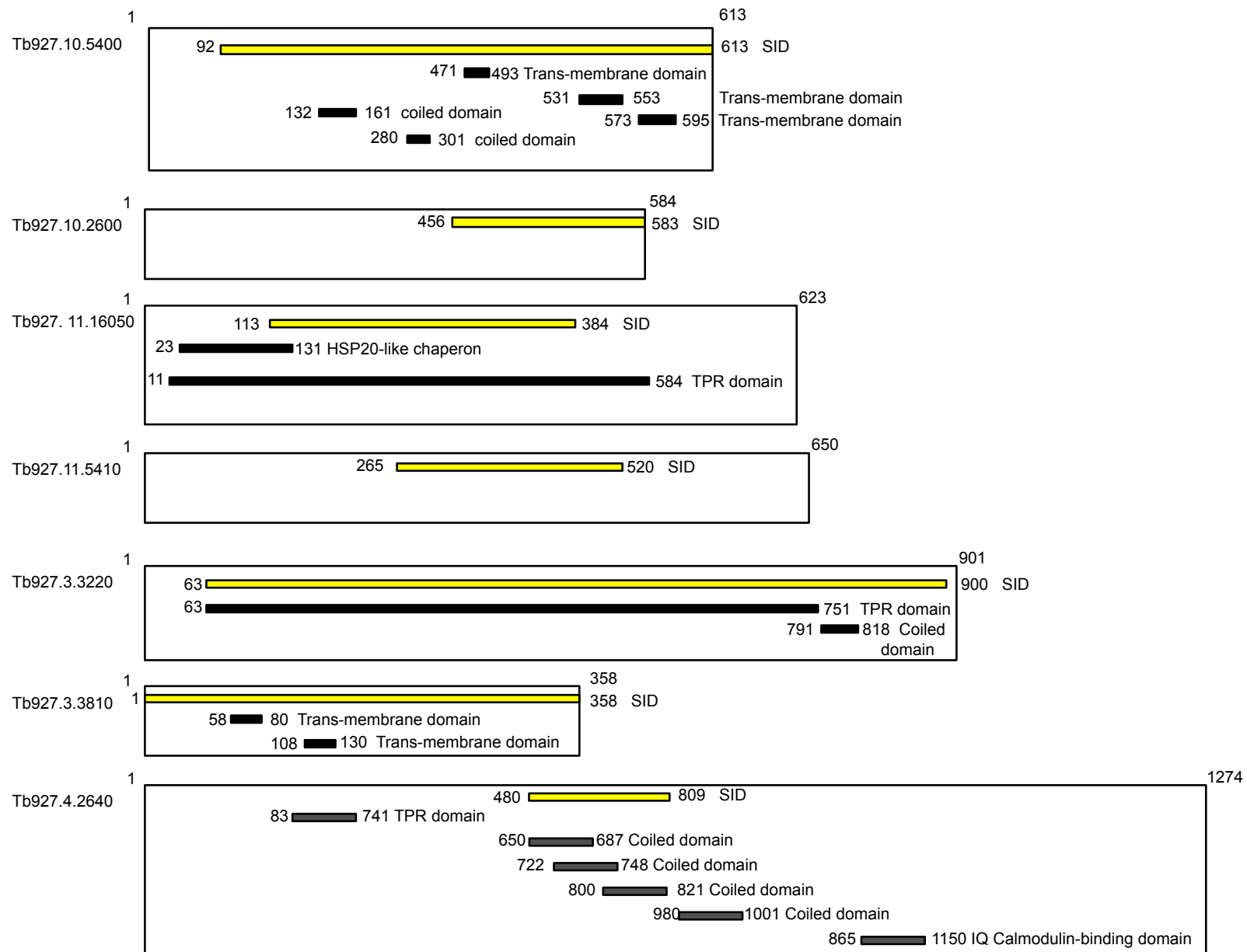

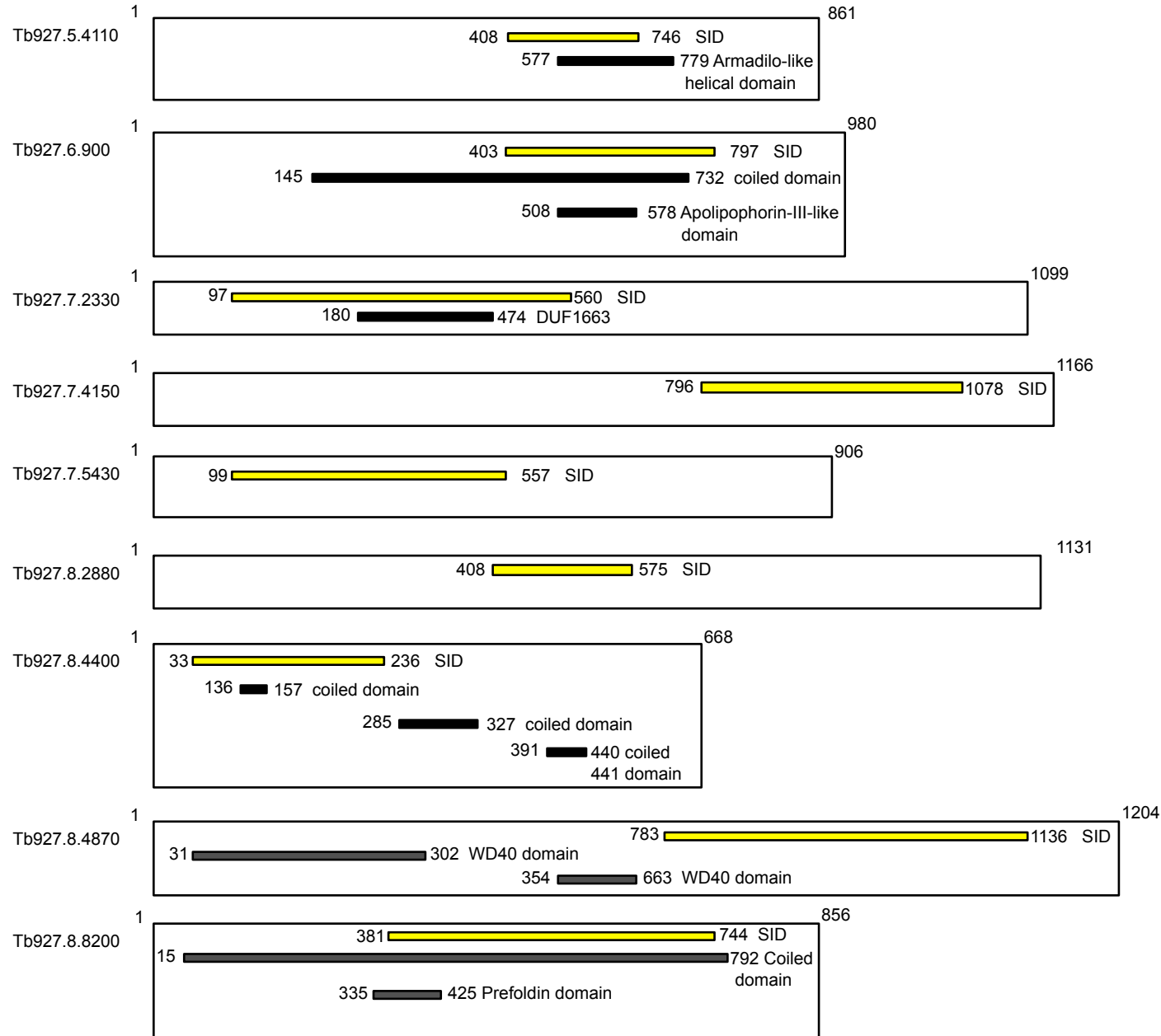

Supplement: Figure S4 — Domain organization of moderate confidence SF3a60 associated proteins. The hits, their corresponding domains and the amino acid portion involved in the interaction with SF3a60 (SID) are shown. (PDF) [file pone.0091956.s004.pdf]
