# Supplementary material for: Proteins Associated with SF3a60 in T. brucei
Source: PLoS One. 2014 Mar 20;9(3):e91956. doi: 10.1371/journal.pone.0091956 (PMC3961280; doi:10.1371/journal.pone.0091956)
Supplement: Figure S5 — The FF domains of PRP40 proteins. The corresponding FF domains of human FBP11 (O75400.2), yeast PRP40 (NP_012913.3) are shown here in bold and starting points marked with an asterisk (*) within the alignment, except for T. brucei FBP11 homologue where they are absent. (PDF) [file pone.0091956.s005.pdf]

Figure S5

		*1
FBP11	(350)	DQSVEVSSNTGEETSKQETVADFTPKKEEEESQ**PAKKTYTWNTKEEAKQA**
PRP40	(103)	---------------EKQEPGRTINEEESQYANNSKLLNV**RRRTKEEAEKE**
Tb927.1.3560	(257)	EYQNRIKFLKEDLENKFNKEKEMLEKQYASETEQVINEFIARSETEKKKH
FBP11	(400)	F**KELLKEKRVPSNASWEQAMKMIIN-DPRYSALAKLS-EKKQAFNAYKVQ**
PRP40	(139)	**FITMLKENQVDSTWSFSRIISELGTRDPRYWMVDDDPLWKKEMFEKYLSN**
Tb927.1.3560	(307)	QLLEEKHNREYSEAKLRWKAEIEALNEQKTKEIEEIKKMDTRLLDAMKQC
		*2
FBP11	(448)	TEKEEK--EEARSKY**KEAKESFQRFLENHEKMTSTTRYKKAEQMFGEMEV**
PRP40	(189)	**RSAD**QL--LKEHNET**SKFKEAFQKMLQNNSHIKYYTRWPTAKRLIADEPI**
Tb927.1.3560	(357)	TEEADKCVSDFISTYEDARNALTRALQGTRETHLTLVEPSLSMLIDRAQQ
FBP11	(496)	**WN--AISERDRLEIYEDVLFFLSKKE-KEQAKQLR**----KRNWEALKNIL
PRP40	(237)	**YKHSVVNEKTKRQTFQDYIDTLIDTQ-KE**SKKKLKTQALKELREYLNGII
Tb927.1.3560	(407)	SYDKAIKDLGACYASESENIKLKFAANIVVAERDAKAAVEEIMRQKENRI
		*3
FBP11	(539)	**DNMANVTYSTTWSEAQQYLMDN-PTFAEDEELQNMDKEDALICFEEHIRA**
PRP40	(286)	TTSSSETFITWQQLLNHYVFDKSKRYMANRHFKVLTHEDVLNEYLKIVNT
Tb927.1.3560	(457)	SFISENTRPTAVIGKNRTETRG—TQCEWDDLEDIQNKHTYAEGTSTVSC
		*4
FBP11	(588)	**LEKEEEE**EKQKSLLRERRR**QRKNRESFQIFLDELHEHGQLHSMSSWMELY**
PRP40	(336)	IENDLQNKLNELRLRNYTR**DRIARDNFKSLLREVPIK--IKANTRWSDIY**
Tb927.1.3560	(505)	NHNADTIQSVKEILGDAVATLRG--TRDEELNELKVHLREFAEEELRTIF
FBP11	(638)	**PTISSDIRFTNMLGQPGSTALDLFKFYVEDLKARYHD**EKKIIKDILKDKG
PRP40	(384)	**PHIKSDPRFLHMLGRNGSSCLDLFLDFVDE**QRMYIFAQRSIAQQTLIDQN
Tb927.1.3560	(553)	DKISQG-R------QP--VSCATIVDTRDDMNVLQHVRPEPVKDEGNAQK
FBP11	(688)	FVVEVN----TTFEDFVAIISSTKRSTTLDAGNIKLAFNSLLEKAEARER
PRP40	(434)	FEWNDADSDEITKQNIEKVLENDRKFDKVDKEDISLIVDGLIKQRNEK**IQ**
Tb927.1.3560	(594)	DSHAPP-VEILRKEDLATAVSEAFERIFVNTPSFPFGANKESTHVEEPDT
		*5
FBP11	(734)	EREKEEARKM**KRKESAFKSMLKQAAPPIELDAVWEDIRERFVKEPAFEDI**
PRP40	(484)	**QKLQNERRILEQK**-------------------------------------
Tb927.1.3560	(643)	PDECRTPSVLGMNAVGLDQQVRGFPISIQDQR---SLLESEIKRVADMRR
FBP11	(784)	**TLESERKRIFKDFMHVLEHECQH**HHSKNKKHSKKSKKHHRKRSRSRSGSD
PRP40	(497)	----------K**HYFWLLLQRTYTKTGKPK--PSTWDLASKELGESLEYKA**
Tb927.1.3560	(690)	IIESQR--TKLEKRRTHLHTTRHRWKQDVVIAKKEGVKASSHQGQLLNKV
FBP11	(834)	SDDDDSHSKKKRQRSESRSASEHSSSAESERSYKKSKKHKKKSKKRRHKS
PRP40	(535)	**LGDEDNIRRQIFEDFKPESSA**PTAESATANLTLTASK--------KRHLT
Tb927.1.3560	(738)	RHVLDGHIKKFEYDEAVLRGSEEWLLMKERSIHKMEHRIRDAERRMVSSG
